# Retrospective analysis of leflunomide and low-dose methylprednisolone for the treatment of diabetic nephropathy combined with membranous nephropathy

**DOI:** 10.3389/fendo.2022.941215

**Published:** 2022-08-30

**Authors:** Shunlai Shang, Shaoyuan Cui, Wenjuan Wang, Chao Wang, Ping Li, Wenge Li, Qinggang Li

**Affiliations:** ^1^ Department of Nephrology, China-Japan Friendship Hospital, Beijing, China; ^2^ State Key Laboratory of Kidney Diseases, Department of Nephrology, The First Medical Center, Chinese PLA General Hospital, National Clinical Research Center for Kidney Diseases, Medical School of Chinese PLA, Chinese PLA Institute of Nephrology, Beijing, China; ^3^ School of Medicine, Nankai University, Tianjin, China; ^4^ Clinical Medical School, Guangdong Pharmaceutical University, Guangzhou, China

**Keywords:** leflunomide, methylprednisolone, diabetic kidney disease, membranous nephropathy, efficacy

## Abstract

Diabetic kidney disease (DKD) combined with Membranous Nephropathy (MN) was observed in some patients with the increasing of Diabetic patients. However, no treatment guidelines are available for DKD combined with MN. In this study, we for the first time analyzed the safety and efficacy of leflunomide (LEF) combined with low-dose glucocorticoid methylprednisolone (MP) in the treatment of DKD with MN. We retrospectively collected the clinical data of patients with the highest number of DKD combined with MN diagnosed by renal biopsy between December 2016 and December 2020. The inclusion criteria were a history of diabetes for more than 20 months, no glucocorticoid therapy or immunosuppressant therapy for at least 6 months, urine protein level greater than 3.5 g, and a follow-up time of 16 months. In addition to conservative treatment, the patients received LEF monotherapy (LEF, n = 38) or LEF combined with low-dose methylprednisolone (LEF+MP, n = 26). After 16 months of treatment, the complete remission rate was 2.6%, and the remission rate was 15.8% in the LEF group; in the LEF+MP group, the complete remission rate and the remission rate were 23.1% and 34.6%, respectively. At month 16, the urine protein level was lower than the baseline value in both groups (*p* < 0.05) and was significantly lower in the LEF+MP group than in the LEF group (*p* < 0.05). Serum albumin levels were higher than the baseline value in both groups (*p* < 0.05), with no significant between-group difference (*p* > 0.05). No inter- or intragroup difference in serum creatinine or glycated hemoglobin was observed. During treatment, the relapse rate was lower in the LEF+MP group than in the LEF group (*p* < 0.05). No irreversible adverse events were observed. In summary, LEF+MP is more effective than LEF monotherapy for DKD combined with MN. Large, long-term, randomized, double-blind, controlled studies are needed to further validate the clinical efficacy of LEF+MP.

## Introduction

With the aging of the population, chronic kidney disease (CKD) has become more prevalent and is a serious threat to public health. In a Chinese study published in 2017, renal biopsy data of 40,759 CKD patients revealed significant changes in the spectrum of CKD from 1997-2002 to 2003-2014. Specifically, diabetic kidney disease (DKD) accounted for 20.76% (up from 6.5%) of secondary glomerular diseases, whereas membranous nephropathy (MN) accounted for 18.42% (up from 9.89%) of primary glomerular diseases (1). Patients with a clinical diagnosis of DKD still experience varying degrees of progressive deterioration after conservative treatment. The increasing use of renal biopsy shows different pathologies in diabetic patients with substantial proteinuria. Renal biopsies have shown that approximately 18.1% of DKD patients have nondiabetic nephropathy, particularly MN (2). Both DKD and MN present substantial proteinuria, with varying degrees of nephrotic syndromes, making it difficult to differentiate and manage them in clinical practice.

Of the primary kidney diseases, MN is one of the main causes of nephrotic syndrome in middle-aged and elderly individuals. MN is characterized by subepithelial deposition of immune complexes. Kidney Disease: Improving Global Outcomes (KDIGO) recommends glucocorticoids combined with an alkylating agent as the first-line treatment for MN. Methylprednisolone (MP) and cyclophosphamide (CTX) are commonly used in the clinic, but they are associated with increased risk of bone marrow suppression, infection, and malignant tumors (3). Leflunomide (LEF) is an immunomodulator with antiproliferative and anti-inflammatory effects (4–6). Compared with no LEF treatment, LEF combined with low-dose glucocorticoids is effective in treating MN, with a remission rate of 71.9% by month 15 (7); compared with the traditional regimen (CTX combined with low-dose glucocorticoids), LEF combined with low-dose glucocorticoids is equally effective for MN, but with fewer side effects (8).

DKD is a microvascular condition caused by a disorder of glucose metabolism disorder. A substantial, abnormal accumulation of glucose metabolites in the kidneys can specifically activate the renin–angiotensin system and immune inflammatory response, leading to thickening of the glomerular basement membrane, an increased mesangial matrix, glomerular sclerosis, tubular damage, and subsequent proteinuria (9–12). After active combined supportive treatment, especially taking ACE inhibitors, the remission rate of proteinuria in diabetic nephropathy was only 26% (13) The 2020 KDIGO guidelines for diabetes management for CKD patients are mainly intended for patients with diabetes and CKD and include recommendations for comprehensive care, lifestyle intervention, blood glucose monitoring, hypoglycemic regimens, and patient management (14, 15). The guidelines do not make specific recommendations for DKD combined with MN.

Clinical evidence has shown that DKD patients should avoid glucocorticoids, while MN patients should receive high-dose glucocorticoids and an immunosuppressant, which may affect glucose utilization, promote gluconeogenesis, and result in uncontrolled blood glucose (16). No consensus has been reached on the treatment of DKD combined with MN, and further research is needed to investigate the effectiveness of low-dose glucocorticoids combined with an immunosuppressant, which would be added to help minimize the impact of glucocorticoids on blood glucose.

We used clinical evidence and a literature review, to retrospectively analyze the effectiveness and safety of LEF combined with low-dose methylprednisolone in patients with DKD combined with MN and propose a new regimen supported by robust evidence to treat this condition.

## Methods

This was a single-center retrospective analysis. The protocol was approved by the Ethics Committee of the PLA General Hospital and China-Japan Friendship Hospital.

### Study population

We collected the clinical data of patients with biopsy-confirmed DKD combined with MN treated at the PLA General Hospital between December 2016 and December 2020.

(1) Inclusion criteria

1) ≥ 18 years of age; 2) a history of diabetes; 3) follow-up ≥ 16 months; 4) no glucocorticoids or immunosuppressive therapy for at least 6 months; 5) urine protein > 3.5 g; and 6) estimated glomerular filtration rate (eGFR) > 50 ml/min/1.73 m2, per the chronic Kidney Disease Epidemiology Collaboration (CKD-EPI) equation.

(2) Exclusion criteria

(1) Other biopsy-confirmed chronic glomerulonephritis; 2) systemic diseases, such as a tumor or connective tissue disease; 3) infectious diseases such as hepatitis B and hepatitis C; 4) inability to take the drugs as prescribed; and 5) pregnant or nursing women.

### Groups and treatments

The patients were divided into two treatment groups:

(1) LEF monotherapy group (LEF): LEF tablets at a dosage of 50 mg/day for 3 days, followed by a maintenance dosage of 20-30 mg/day for 6 months, and then additional 3 months for patients with complete remission (CR) or partial remission (PR) or 12 months for nonresponders. The dosage could be adjusted based on treatment outcome.

(2) LEF combined with low-dose methylprednisolone group (LEF+MP): LEF was taken in the same manner as in the LEF group. Methylprednisolone was taken at a dosage of 0.5-0.8 mg/kg/day for 2 months, followed by tapering (5 mg every 1-2 months) if urine protein levels improved. The dosage could be adjusted based on patient conditions, tolerability, and treatment outcome.

### Outcome measures

(1) Demographics: Sex, age, course of diabetes, a history of hypertension, and medications.

(2) Clinical data: Laboratory tests such as glycosylated hemoglobin (HbA1c), serum albumin (ALB), urine protein, serum creatinine (SCr), and serum phospholipase A2 receptor (PLA2R). The anti-PLA2R level were measured using published and validated methods produced by EUROIMMUN AG (Germany), an enzyme-linked immunosorbent assay (ELISA), which were performed according to the instructions of the manufacturer. The range of serum anti-PLA2R antibody could be 2 to 1500 RU/mL, and the results were recorded as positive when the concentration was >20 RU/mL. renal biopsy to assess the presence of glomerulosclerosis, segmental sclerosis and interstitial fibrosis/tubular atrophy (IFTA), and the number of renal arterial lesions (arteriole hyalinosis and renal arteriole sclerosis).

(3) Follow-up: Once a month for at least 16 months to establish a comprehensive follow-up database.

(4) Clinical visits: Laboratory tests such as HbA1c, ALB, urine protein, and SCr at each visit.

### Efficacy evaluation

(1) Primary efficacy evaluation:Remission rates and complete remission rate. CR, complete remission; PR, partial remission; remission includes complete and partial remission.

(2) Secondary efficacy evaluation: urine protein (g/d), ALB, and SCr.

(3) Efficacy criteria:

1) CR: Urine protein < 0.3 g/d at least twice a week; normal ALB and SCr.

2) PR: Urine protein < 3.5 g/d or ≥ 50% lower than the peak; ALB higher than or the same as baseline; SCr level lower than or the same as baseline.

3) Time to remission: Time to initial CR or PR.

4) Nonresponders: Patients who did not meet any of the above criteria.

5) Relapse: After remission, urine protein > 3.5 g/d and ≥ 50% higher than trough concentrations.

6) Renal insufficiency: SCr level ≥ 50% higher than baseline.

### Safety evaluation

Adverse events: Common side effects of LEF include neurological abnormalities (dizziness, decreased response, confusion, difficulty thinking) and gastrointestinal reactions (nausea, vomiting). Methylprednisolone, a glucocorticoid, may cause uncontrolled blood glucose, liver dysfunction, infection, osteoporosis, eyelid swelling, elevated intraocular pressure, and menstrual disorders (women).

## Statistical analysis

SPSS v17.0 was used for data processing and statistical analysis. Normally distributed continuous variables are expressed as the mean± standard deviation (SD) and were analyzed with Student’s t-test. Nonnormally distributed continuous variables are expressed as the median (interquartile range) and were analyzed with the nonparametric rank sum test. Categorical variables are expressed as percentages and were analyzed with Fisher’s exact test or Pearson’s χ^2^ test. The probability of remission was analyzed using Kaplan-Meier curves, and differences were estimated by using the log-rank test.

## Results

### Baseline characteristics

**Figure 1:**
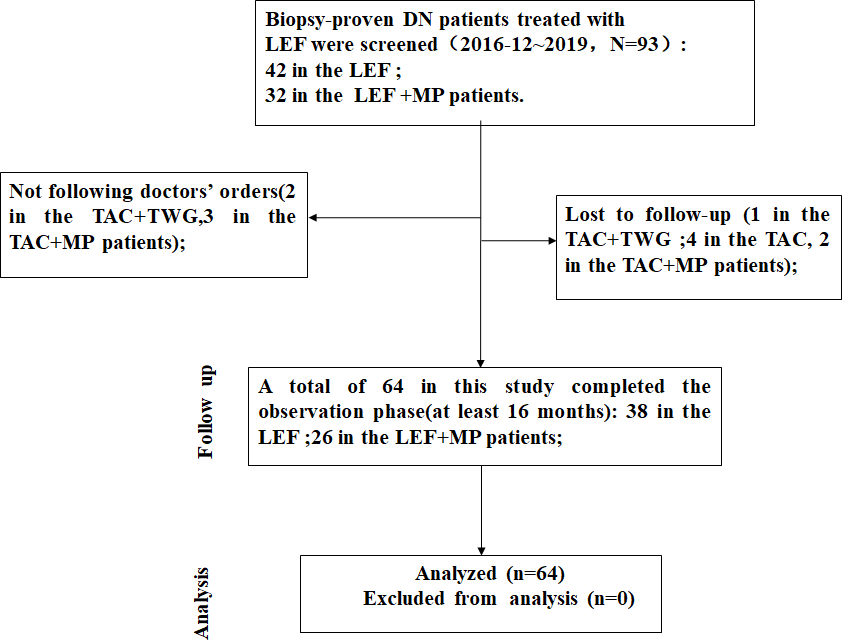
The screening process. We collected the clinical data of 74 patients with biopsy-confirmed DKD combined with MN. Ultimately, a total of 64 patients completed the study, 38 in the LEF group and 26 in the LEF+MP group.

We collected the clinical data of 74 patients with biopsy-confirmed DKD combined with MN between December 2016 and December 2019, consisting of 42 patients in the LEF group and 32 patients in the LEF+MP group. A total of 64 patients completed the study, 38 in the LEF group and 26 in the LEF+MP group. Six patients (LEF: n = 3, LEF+MP: n = 3) were lost to follow-up, and four patients did not take the drugs as prescribed (LEF: n = 1, LEF+MP: n = 3). The screening process is shown in **Figure 1**.

We collected the patients’ clinical data, including demographics, clinical diagnosis, clinical exams, and laboratory tests. No significant between-group difference was observed in the baseline data (*p* < 0.05) (**Table 1**).

**Table 1:** Baseline characteristics of the patients in the two groups.

Characteristics	LEF group (n=38)	LEF + MP group (n=26)	*P* value
Sex (male/female)	23/15	19/7	0.229
Age (years)	59 (13)	59 (11.5)	0.805
Diabetes duration (years)	14.5 (4)	13 (5)	0.328
Biguanides/Thiazolidinones/ α- Glucosidase inhibitor/Sulfonylureas	9/12/8/9	7/8/5/6	0.992
Systolic BP (mmHg)	138 ± 13	136 ± 20	0.234
Diastolic BP (mmHg)	71 ± 8	80 ± 10	0.934
Daily urinary protein (g/24 h)	5.029 (1.53)	4.805 (3.05)	0.608
Serum albumin (g/L)	25.65 (6.55)	24.15 (7.1)	0.805
Anti-PLA2R (positive/negative)	20/18	14/12	0.924
Scr (μmol/L)	72.3 (17.38)	74.95 (16.72)	0.610
HbA1c (%)	6.7 (1.83)	6.35 (1.2)	0.427
Glomerular sclerosis, %	8 (6.25)	13 (8)	0.187
Segmental sclerosis, n (%)	8 (21.1)	6 (23.1)	0.847
IFTA, n (%)	31 (81.58)	20 (76.92)	0.649
Afferent arterial, n (%)	34 (94.44)	24 (92.31)	1

HbA1c, glycosylated hemoglobin; ALB, serum albumin; SCr, serum creatinine; Anti-PLA2R, serum phospholipase A2 receptor); IFTA, interstitial fibrosis/tubular atrophy.

### Response to therapy

(1) Primary efficacy evaluation

**Figure 2:**
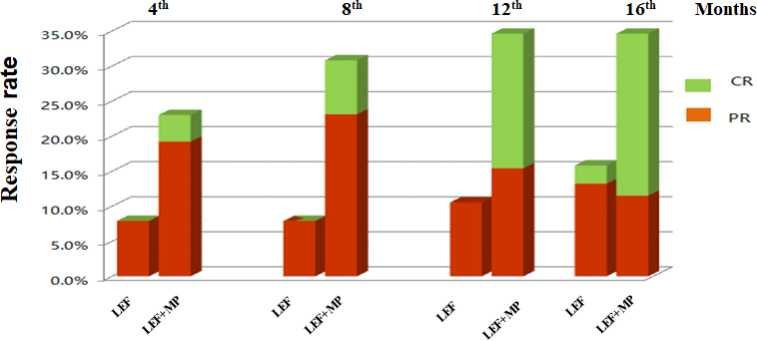
Remission rates (CR + PR, CR, PR) in the LEF group and the LEF+MP group over time. Percentage of complete (green) and partial (red) remissions in the two groups.CR, complete remission; PR, partial remission.

**Figure 3:**
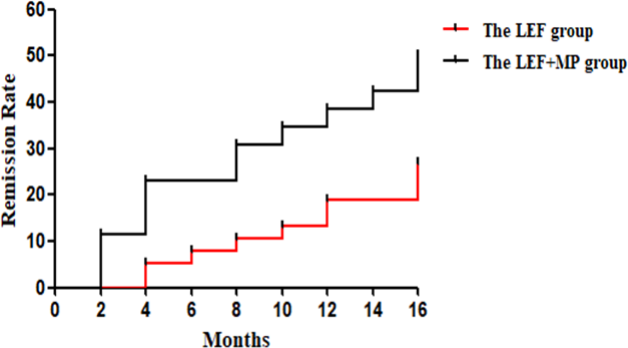
The log-rank test in the LEF group and the LEF+MP group. Compared with the LEF group, the LEF+MP group had a higher remission rate (log-rank, *p* < 0.05) (**Figure 3**).

At month 16, the remission rate was 15.8% in the LEF group (n = 38), which was significantly lower than that in the LEF+MP group (n = 26; 34.6%, *p < 0.05). In the LEF group, the PR rate gradually improved to 7.9% at 4 months, 7.9% at 8 months, 10.5% at 12 months, and 13.2% at 16 months. CR did not occur until month 16 (2.6%). In the LEF+MP group, the PR rate was 19.28% at 4 months, 19.2% at 8 months, 15.4% at 12 months, and 11.5% at 16 months. The CR rate gradually improved: 3.8% at month 4, 7.7% at month 8, 19.2% at month 12, and 23.1% at month 16. See **Table 2** and **Figure 2** for details. The LEF+MP group had a higher remission rate than the LEF group (log-rank, *p* < 0.05) (**Figure 3**).

**Table 2:** *P* value and remission rates of the LEF + MP group compared with the LEF group.

Project	Month	LEF group (n=38)	LEF+ MP group (n=26)	*P*
CR + PR	4^th^	7.9%	23.08%	0.177
	8^th^	7.9%	26.9%	0.041
	12^th^	10.5%	34.6%	0.019
	16^th^	15.8%	34.6%	0.041
CR	4^th^	0%	3.8%	0.406
	8^th^	0%	7.7%	0.161
	12^th^	0%	19.2%	0.019
	16^th^	2.6%	23.1%	0.030

(2) Secondary efficacy evaluation

**Figure 4:**
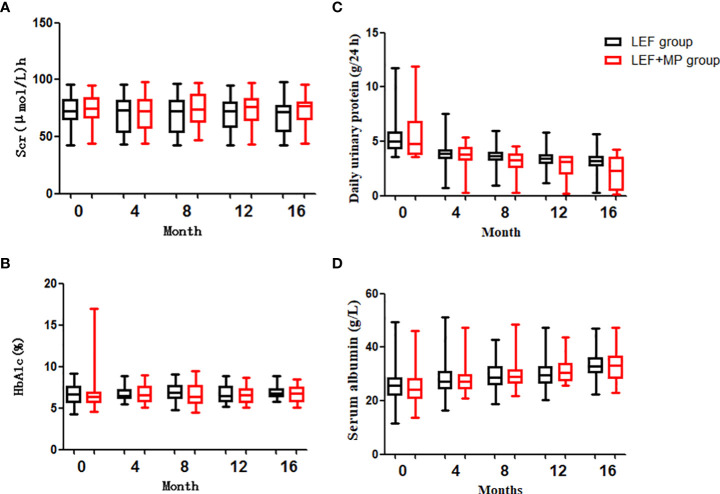
Clinical indicators (SCr, HbA1c, daily urine protein, serum albumin). **(C)** shows that the decrease in proteinuria was significantly greater in the TAC+TMG group than in the LEF group at months 12^th^ and 16^th^; **(A, B, D)** show that there were no significant differences in SCr, HbA1c, daily urine protein, or serum albumin levels in the LEF group and the LEF+MP group (*P*>0.05).

In both groups, the 24-hour urine protein concentration decreased (p < 0.05) and ALB levels significantly increased (*p* < 0.001) from baseline to month 16, with no significant changes in SCr, HbA1c and Serum albumin *(p* > 0.05);and you could see **Figures 4A, B, D**. At month 16, the 24-hour urine protein concentration was 2.275 (2.99) g/d in the LEF+MP group and 3.2 (0.79) g/d in the LEF group, indicating the superior efficacy of combination therapy (*p* < 0.05), as shown in **Figure 4C**. No significant between-group difference was observed in other indicators (*p* > 0.05). See **Table 3** for details,

**Table 3:** Urine protein, ALB, SCr, and HbA1c at each time point in the LEF group and the LEF+MP group, with *P* values.

Project	Month	LEF group (n=38)	LEF+ MP group (n=26)	*P^#^*	*P^a^*	*P^b^*
Daily urinary protein (g/24 h)	4	3.9 (0.8)	3.825 (1.19)			
	8	3.65 (0.67)	3.3 (1.21)			
	12	3.425 (0.78)	3.11 (1.58)			
	16	3.2 (0. 79)	2.275 (2.99)	0.013	<0.001	<0.001
Serum albumin (g/L)	4	27.25 (6.43)	27.25 (5.07)			
	8	28.8 (6.58)	28.9 (5)			
	12	29.5 (6.05)	30.6 (6.4)			
	16	32.75 (5.6)	33.2 (8.18)	0.879	<0.001	<0.001
Scr (μmol/L)	4	73.5 (28.25)	72.8 (24.75)			
	8	72.2 (27.55)	73.9 (23.78)			
	12	72.8 (21.3)	76.35 (18.52)			
	16	71.5 (21.9)	77.25 (15.23)	0.196	0.496	0.805
HbA1c (%)	4	6.5 (1)	6.6 (1.73)			
	8	6.9 (1.5)	6.35 (2.08)			
	12	6.5 (1.73)	6.6 (1.58)			
	16	6.75 (0.87)	6.8 (1.65)	0.686	0.208	0.355

P^#^, The LEF group versus the LEF+MP group; P^a^, versus baseline, LEF group; P^b^, versus baseline, LEF+MP group.

(3) Time to remission

The time to remission was 10.200 ± 5.533 months in the LEF group and 7.846 ± 5.321 months in the LEF+MP group, and there was no significant difference between the two groups. (*p* > 0.05).

(4) Relapse

In the LEF treatment group, nine patients responded to the treatment and one relapsed (11.1%). In the LEF+MP group, 13 patients responded to the treatment and one relapsed (7.7%). The relapse rate was significantly lower in the LEF+MP treatment group (*p* < 0.05).

### Adverse events

Among the 64 patients who completed this study, 13 experienced adverse events (LEF: n=4, 10.5%; LEF+MP: n=9, 34.6%), with no significant between-group difference (*p* > 0.05). All adverse events were mild and were alleviated after symptomatic care. See **Table 4** for details.

**Table 4:** Adverse events among the two groups.

Adverse event	LEF group	LEF + MP group
Anemia	1 (2.6%)	0 (0%)
Abnormal liver function	1 (2.6%)	1 (3.8%)
Infection	1 (2.6%)	2 (7.7%)
Gastrointestinal symptoms	1 (2.6%)	1 (3.8%)
Eye disease	0 (0%)	1 (3.8%)
Osteoporosis	0 (0%)	2 (7.7%)
menstrual disorders	0 (0%)	1 (3.8%)

## Discussion

With the aging of the population, diabetes is becoming more prevalent, as is DKD. In a pathology study on DKD, 0.8% to 23.0% (mean: 12%) of the patients had both DKD and nondiabetic nephropathy. These patients have a lower survival rate and are more likely to progress to end-stage renal disease(ESRD) than those with DKD or nondiabetic nephropathy only (17). Therefore, renal biopsy should be considered for DKD patients with atypical manifestations such as nondiabetic retinopathy, a short course of disease, or nonnephrotic proteinuria who do not respond to conservative treatment. This provides a histopathological basis for the diagnosis and effective specific immunotherapy of DKD comorbid with nondiabetic nephropathy.

At present, the treatment of diabetic nephropathy is mainly to reduce blood pressure, hypoglycemic treatment, the effect is not very ideal. Now many studies have found that diabetes is also an immune disease, suggesting immune intervention. This study uses immunosuppressive therapy, which is also an exploration of immunointerventional therapy (18).

No guidelines are available for the treatment of DKD combined with MN, which is a more complex condition. Glucocorticoids and/or immunosuppressants have been used less often in patients with DKD and MN than for patients with MN only (50.0% vs 23.1% *p* < 0.05). After 6 months of monotherapy with a calcineurin inhibitor, the CR rate was 33.3% in the MN group, which was significantly higher than that in the DKD + MN group (0.0%) (16), suggesting the importance of starting combination therapy early with glucocorticoids and immunosuppressants. In this study, we focused on the treatment outcome of low-dose glucocorticoids combined with an immunosuppressant in patients with DKD+MN.

In our study, the remission rate of LEF+MP group was 19.2% at 12 months and 23.1% at 16 months, which was not significantly different from the 26% remission rate of diabetic nephropathy studied by Hovind P et al (13). In addition, the remission rate of THE LEF+MP group was significantly higher than that of the LEF group, and the recurrence rate of the LEF+MP group was lower, indicating that the treatment effect of the LEF+MP group was better than that of the LEF group, which has certain clinical significance. LEF is an effective immunomodulator for immune-mediated kidney diseases such as MN, systemic lupus erythematosus, IgA nephropathy, and refractory nephrotic syndrome (19–21).Compared with no LEF treatment, LEF combined with oral glucocorticoids enables a higher remission rate in MN patients (7). LEF inhibits osteopontin (OPN)/transforming growth factor beta 1 (TGF-β1)-mediated extracellular matrix deposition, tubular interstitial fibrosis, and tubular epithelial–mesenchymal transdifferentiation to reduce kidney injury in diabetic rats (6). Moreover, LEF protects the kidneys by inhibiting the activation of NF-κB and reducing the expression of cytokines and adhesion factors (4).

LEF has fewer and milder side effects than CTX and other immunosuppressants, with common side effects of nausea, vomiting, and liver damage, all of which can be corrected with careful monitoring and proper management. Many studies have found that patients with DKD should avoid using glucocorticoids, which may affect glucose utilization, promote gluconeogenesis and lead to uncontrolled blood glucose (16). This study showed no significant difference in hba1c between leflunomide combined with low-dose glucocorticoid and leflunomide alone, indicating that low-dose glucocorticoid has the least effect on blood glucose in DKD patients with MN. In summary, LEF combined with low-dose MP is an inexpensive treatment with few side effects and may become a new regimen for patients with DKD combined with MN.

The number of patients collected in this study is larger than the number of cases reported by Bhadauria D (22) and Qian Y, et al. (16), which can better represent the current treatment effect and side effects of this disease. We hope that this study can provide reference for future treatment of this disease.

As a retrospective analysis, this study has certain limitations. First, it was a single-center study with a small sample size and a short follow-up period. Second, we did not include a positive control group given the canonical MN regimen (glucocorticoids + calcineurin inhibitor). Moreover, we did not perform stage-specific subgroup analysis. Large, multicenter, randomized, prospective studies are needed to further validate the results.

## Conclusion

We retrospectively collected the clinical data of patients with the highest number of DKD combined with MN diagnosed by renal biopsy, and firstly analyzed the safety and effectiveness of LEF combined with low-dose MP for the treatment of this disease. For this patient group with complex DKD, compared with immunosuppressive therapy alone, immunosuppressants combined with low-dose glucocorticoids has a better effect. It is hoped that our study provides a reference for future clinical treatment of this disease.

## Data availability statement

The original contributions presented in the study are included in the article/supplementary material. Further inquiries can be directed to the corresponding authors.

## Ethics statement

The studies involving human participants were reviewed and approved by the Chinese PLA General Hospital Medical Ethics Committee. The patients/participants provided their written informed consent to participate in this study.

## Author contributions

QL designed the study. SS and SC wrote the original draft. SS, WW, CW, and QL provided the patient samples and the corresponding clinical data. SS, WW, SC, and PL validated and interpreted the data, WL played an important role in revising the manuscript. All authors contributed to the article and approved the submitted version.

## Funding

This work was supported financially by National Natural Science Foundation of China (81770664).

## Acknowledgments

We are grateful to all of the patients for their participation in this study.

## Conflict of interest

The authors declare that the research was conducted in the absence of any commercial or financial relationships that could be construed as a potential conflict of interest.

## Publisher’s note

All claims expressed in this article are solely those of the authors and do not necessarily represent those of their affiliated organizations, or those of the publisher, the editors and the reviewers. Any product that may be evaluated in this article, or claim that may be made by its manufacturer, is not guaranteed or endorsed by the publisher.
